# Understanding the Role of Orexin Neuropeptides in Drug Addiction: Preclinical Studies and Translational Value

**DOI:** 10.3389/fnbeh.2021.787595

**Published:** 2022-01-20

**Authors:** Alessandra Matzeu, Rémi Martin-Fardon

**Affiliations:** Department of Molecular Medicine, The Scripps Research Institute, La Jolla, CA, United States

**Keywords:** orexin, cocaine, alcohol, opioids, nicotine

## Abstract

Orexins (also known as hypocretins) are neuropeptides that participate in the regulation of energy metabolism, homeostasis, sleep, feeding, stress responses, arousal, and reward. Particularly relevant to the scope of the present review is the involvement of the orexin system in brain mechanisms that regulate motivation, especially highly motivated behavior, arousal, and stress, making it an ideal target for studying addiction and discovering treatments. Drug abuse and misuse are thought to induce maladaptive changes in the orexin system, and these changes might promote and maintain uncontrolled drug intake and contribute to relapse. Dysfunctional changes in this neuropeptidergic system that are caused by drug use might also be responsible for alterations of feeding behavior and the sleep-wake cycle that are commonly disrupted in subjects with substance use disorder. Drug addiction has often been associated with an increase in activity of the orexin system, suggesting that orexin receptor antagonists may be a promising pharmacological treatment for substance use disorder. Substantial evidence has shown that single orexin receptor antagonists that are specific to either orexin receptor 1 or 2 can be beneficial against drug intake and relapse. Interest in the efficacy of dual orexin receptor antagonists, which were primarily developed to treat insomnia, has grown in the field of drug addiction. Treatments that target the orexin system may be a promising strategy to reduce drug intake, mitigate relapse vulnerability, and restore “normal” physiological functions, including feeding and sleep. The present review discusses preclinical and clinical evidence of the involvement of orexins in drug addiction and possible beneficial pharmacotherapeutic effects of orexin receptor antagonists to treat substance use disorder.

## Introduction

Since their discovery in 1998 (de Lecea et al., 1998; Sakurai et al., 1998a), orexins (Orxs) have received growing research interest. Orexins are neuropeptides that have been shown to regulate a range of physiological and behavioral functions, including energy metabolism, homeostasis, arousal, sleep, and feeding (Sutcliffe and de Lecea, 2000; Mieda and Yanagisawa, 2002). Particularly important for the scope of this review is the role of these neuropeptides in modulating stress (Berridge et al., 2010) and reward-motivated states (Harris et al., 2005; Martin-Fardon et al., 2010, 2016), components that have a strong impact on drug addiction. Independently discovered in 1998 by two independent groups (de Lecea et al., 1998; Sakurai et al., 1998a), orexin A (OrxA) and orexin B (OrxB), also referred to as hypocretin 1 (Hcrt1) and hypocretin 2 (Hcrt2), are neuropeptides that derive from a common precursor, prepro-orexin, that is produced exclusively in well-defined subregions of the hypothalamus (HYP). An estimated 3,000–6,700 neurons express Orx in the rat brain, and 50,000–80,000 neurons express Orx in the human brain (Thannickal et al., 2000; Fronczek et al., 2005; Modirrousta et al., 2005; de Lecea, 2015; Soya and Sakurai, 2020), distributed in the lateral HYP (LH), dorsomedial HYP (DMH), and perifornical area (PFA; de Lecea et al., 1998; Sakurai et al., 1998a).

Two isoforms of G protein-coupled receptors have been identified as molecular targets for Orxs, orexin receptor 1 (OrxR1 [Hcrt-r1]) and orexin receptor 2 (OrxR2 [Hcrt-r2]; Sakurai et al., 1998a). OrxR1 binds OrxA with 20–30 nM affinity but has much lower affinity (10- to 1,000-fold lower) for OrxB, whereas OrxR2 binds both peptides with similar affinity (in the 40 nM range; Sakurai et al., 1998b; Scammell and Winrow, 2011). Both OrxRs are G protein-coupled receptors. OrxR1 was initially thought to couple exclusively G_q_ protein, and OrxR2 was thought to couple G_q_ and G_i/o_ proteins (Sakurai et al., 1998b; Smart et al., 1999; Lund et al., 2000; Holmqvist et al., 2002), but more recent evidence suggests that OrxR signaling is significantly more diverse. In fact, OrxRs are able to couple members of at least three G-protein families and other proteins, through which they regulate non-selective cation channels, phospholipases, adenylyl cyclase, and protein and lipid kinases (e.g., Kukkonen and Leonard, 2014). These two OrxRs are extensively distributed throughout the brain (Marcus et al., 2001), which can explain intricate participation of the Orx system in regulating several physiological functions. In the brain, OrxR1 and OrxR2 exhibit mostly distinctive expression patterns, with some overlap (Marcus et al., 2001).

One important consideration is that the Orx system plays a pivotal role in several addiction-related behaviors (for review, see Aston-Jones et al., 2010; Mahler et al., 2012; Matzeu and Martin-Fardon, 2020b). The first evidence of the involvement of the Orx system in drug addiction was reported almost two decades ago. These studies demonstrated the involvement of LH Orx neurons in morphine-related behaviors, including drug seeking, dependence, and withdrawal (Georgescu et al., 2003; Harris et al., 2005), opening new perspectives into investigating neuronal mechanisms that are involved in the etiology of drug addiction. Since then, the body of scientific literature on the involvement of the Orx system in drug addiction has grown continuously, including its involvement in cocaine, alcohol, opioid, and nicotine addiction.

Drug addiction has been associated with alterations of the number of Orx neurons (James et al., 2019; Sharma et al., 2020; Matzeu and Martin-Fardon, 2021) and *Orx* gene expression (Sharma et al., 2020). In humans, chronic substance use has been shown to affect nutritional status and eating habits (Rush et al., 2016; Cusack et al., 2021; Mahboub et al., 2021) and cause sleep disturbances (Brooks et al., 2021; Frers et al., 2021; Miller et al., 2021). Therefore, pharmacological manipulations of the Orx system by targeting OrxRs may be a promising strategy to reduce drug intake, mitigate relapse vulnerability, and normalize feeding and sleep. The present review discusses preclinical studies that used animal models of operant behavior and clinical evidence of the involvement of the Orx neuropeptides in drug addiction and the possible beneficial pharmacotherapeutic effects of OrxR antagonists to treat substance use disorder.

Most studies that examined the role of the Orx system in drug addiction only used male subjects. If both males and females were used, then the studies were simply inclusive of both sexes and did not necessary study sex differences. Importantly, however, studies have revealed sexual dimorphism in Orx activity (for review, see Grafe and Bhatnagar, 2020). Sex differences in *Orx* and *OrxR1/R2* mRNA both peripherally and centrally have been documented in preclinical studies (Taheri et al., 1999; Johren et al., 2001, 2002). Female rats exhibit higher levels of *Orx* mRNA in the HYP and higher levels of OrxA in cerebrospinal fluid compared with males (Johren et al., 2002). Moreover, in female rats, Orx-positive neurons show greater activation compared with males (Grafe et al., 2017). Also, OrxR1 and OrxR2 expression appears to be higher in females compared with males, at least in some brain areas (Johren et al., 2002; Loewen et al., 2017). Although only a few clinical studies are available about sex differences in the Orx system, they suggest that women exhibit higher levels of Orx in the brain compared with men (Wennstrom et al., 2012; Schmidt et al., 2013; Lu et al., 2017). Understanding the interplay between sex, Orx, and addiction needs to be addressed, particularly when considering treatments for substance use disorder.

## Neuroadaptations of the Orexin System Induced by Drugs of Abuse

### Cocaine

Prolonged cocaine consumption induces significant alterations of the number of Orx neurons in the HYP and it has been shown that chronic cocaine use increases the number of Orx-expressing cells (James et al., 2019; Matzeu and Martin-Fardon, 2021). These studies used two different models to induce addiction-like behavior: extended access to cocaine self-administration for 6 h/day for 21 days (Matzeu and Martin-Fardon, 2021) and intermittent access to cocaine self-administration for 5 min, followed by 25 min of cocaine non-availability (repeated 12 times/day for 14 days; James et al., 2019). The addiction-like behavioral phenotype that is induced by these animal models was accompanied by persistent increases in the number of Orx-immunoreactive hypothalamic neurons starting at 14–21 days and up to 150 days following the last cocaine exposure (James et al., 2019; Matzeu and Martin-Fardon, 2021). Importantly, this increase in expression was observed exclusively for Orx and not for other neuropeptides, such as melanin-concentrating hormone, in the same hypothalamic region (James et al., 2019). A study investigated the participation of Orx transmission in the posterior paraventricular nucleus of the thalamus (pPVT) in cocaine-related behaviors, showing that exposure to cocaine under extended access conditions increased OrxR2 immunoreactivity at 14–21 days of abstinence, which returned to basal levels after 35 days (Matzeu and Martin-Fardon, 2021). This observation was particularly important because it confirmed the participation of OrxR2 signaling in the pPVT in mediating the priming effect of intra-pPVT OrxA-induced cocaine-seeking behavior (Matzeu et al., 2016; Matzeu and Martin-Fardon, 2021). The persistent upregulation of Orx peptide during abstinence from cocaine, together with alterations of OrxR2 in the pPVT, suggests that cocaine compromises the Orx system, and this effect persists into abstinence and most likely affects brain regions beyond the pPVT that receive HYP Orx projections.

### Alcohol

Similar to cocaine, alcohol consumption has been shown to dysregulate the Orx system, but chronic alcohol intake data have been somewhat controversial. For example, chronic home cage alcohol drinking was shown to reduce *Orx* mRNA expression in the HYP, whereas acute oral alcohol administration by gavage was associated with an increase in *Orx* expression (Morganstern et al., 2010). Moreover, alcohol dependence downregulated *Orx* mRNA expression at 12 h of withdrawal in rats that were made dependent *via* repeated intragastric (i.e., gavage) alcohol administration (Sharma et al., 2020). Consistent with these data, a clinical study reported that blood OrxA levels in patients with alcohol use disorder were inversely related to the severity of withdrawal symptoms (i.e., stronger withdrawal symptoms were associated with lower OrxA expression; Bayerlein et al., 2011). This observation might partially account for the exacerbation of lethargy during the day that is experienced by subjects who suffer from alcohol use disorder during alcohol withdrawal (for review, see Miller et al., 2021).

Opposing results were recently found in our study that investigated the participation of the pPVT in alcohol seeking. We found a significant increase in *Orx* mRNA expression in the HYP and *OrxR1* and *OrxR2* mRNA expression in the pPVT following extinction at 8 h of withdrawal in rats that were made alcohol dependent by chronic intermittent alcohol vapor exposure (Matzeu and Martin-Fardon, 2020a). Consistent with our findings, the Orx system was reported to be upregulated in adult rats following binge-like patterns of alcohol intoxication during adolescence (Amodeo et al., 2020) and in rats that exhibited high novelty-induced locomotor activity, a predictor of high alcohol consumption (Barson et al., 2013). Interestingly, pre-fertilization maternal alcohol consumption in zebrafish significantly increased the number of Orx neurons and alcohol consumption in offspring (Collier et al., 2020). The dependence induction procedure, time point of withdrawal, age of the animals, species, and other experimental manipulations might account for discrepancies in these preclinical studies.

### Opioids

The dysregulation of Orx neuropeptides has also been demonstrated following opioid abuse. An increase in the number of Orx-producing neurons was found in postmortem brains from individuals with heroin addiction (Thannickal et al., 2018). Similar increases in Orx-producing cells were induced in wildtype mice by long-term morphine administration that lasted up to 8 weeks after morphine cessation (Thannickal et al., 2018). Moreover, intermittent access to fentanyl was associated with an increase in the number Orx cells in the HYP (Fragale et al., 2021), suggesting that a common effect of chronic opioid use is the upregulation of Orx-producing cells.

### Other Drugs of Abuse

The downregulation of *Orx* gene expression in blood was reported in nicotine-dependent cigarette smokers and subjects with Δ^9^-tetrahydrocannabinol dependence (Rotter et al., 2012), suggesting that Orx system dysregulation is a common factor across several classes of drugs of abuse.

## Role of Orexin Neuropeptides in Drug Intake

### Cocaine

Orexin neuropeptides facilitate drug-directed behavior, especially under motivationally salient, high-effort conditions (Borgland et al., 2009; Mahler et al., 2014). Particularly significant is a study that used a short-hairpin RNA-encoding adeno-associated viral vector to knock down *Orx* expression throughout the HYP in adult rats and found lower motivation for cocaine intake under both progressive-ratio (PR) and fixed-ratio (FR) schedules in rats that were given extended access to cocaine self-administration. Interestingly, knocking down *Orx* also reduced the intake of a palatable food reward (i.e., sweetened condensed milk). Importantly, *Orx* silencing did not affect food or water consumption and had no effect on general measures of arousal or stress reactivity (Schmeichel et al., 2018). These findings support the hypothesis that Orx neuropeptides promote operant responding for both drug and non-drug rewards, specifically under conditions that require a high degree of motivation (e.g., PR schedules, extended access to cocaine, and a highly palatable food reward).

Much evidence indicates that OrxR1 is critical for driving highly motivated responding for cocaine, particularly in rats with high motivation for the drug. The blockade of OrxR1 by systemic SB334867 administration decreased cocaine self-administration in rats under limited access conditions under an FR5 timeout 20 s (TO20 s) schedule of reinforcement (Hollander et al., 2012) or PR schedule of reinforcement (Hollander et al., 2012; Prince et al., 2015) but did not alter food self-administration under an FR5 TO20 s schedule of reinforcement (Hollander et al., 2012). OrxR1 knockout mice self-administered significantly less cocaine than wildtype mice (Hollander et al., 2012). Another study described the lack of an effect of peripheral OrxR1 and OrxR2 antagonist administration on reducing cocaine self-administration under an FR1 TO20 s schedule in rats with limited access to cocaine (Smith et al., 2009). These findings suggest that Orx signaling is unnecessary for already established cocaine self-administration under low-effort conditions (e.g., low FR combined with short cocaine self-administration sessions). The blockade of OrxR1 preferentially reduced cocaine self-administration under high-effort conditions (e.g., low cocaine dose and PR schedule; Brodnik et al., 2015), in rats with extended access to cocaine (Schmeichel et al., 2017), and in rats that were given intermittent access to cocaine and exhibited an addiction-like phenotype (James et al., 2019). These data demonstrate that OrxR1 plays an important role in regulating the reinforcing and reward-enhancing properties of cocaine and that Orx transmission is important for establishing and maintaining cocaine intake under high motivation conditions.

**FIGURE 1:**
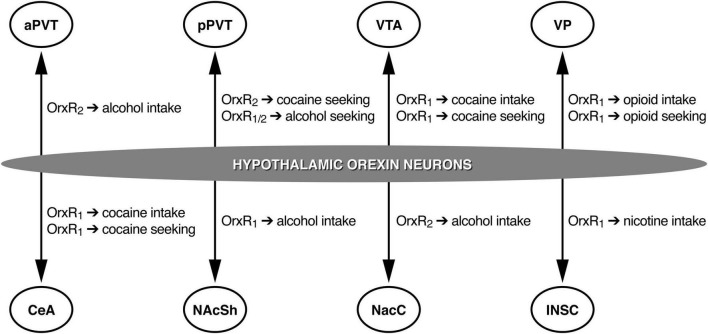
Schematic representation of the roles of different OrxR subtypes in different brain regions. aPVT, anterior paraventricular nucleus of the thalamus; pPVT, posterior paraventricular nucleus of the thalamus; VTA, ventral tegmental area; VP, ventral pallidum; CeA, central nucleus of the amygdala; NAcSh, nucleus accumbens shell; NAcC, nucleus accumbens core; INSC, insular cortex. Arrows represent Orx projections.

Brain regions need to be identified where Orx neuropeptides can mediate drug-directed behavior. For example, Orx inputs to the ventral tegmental area (VTA) are particularly interesting. The majority of Orx axons in the VTA are passing fibers, with only a small portion of Orx fibers in direct synaptic contact on VTA dopamine and γ-aminobutyric acid neurons (Balcita-Pedicino and Sesack, 2007). Intra-VTA OrxA increases the firing rate of VTA dopamine neurons (Korotkova et al., 2003; Muschamp et al., 2007) and increases extracellular dopamine levels in the prefrontal cortex (PFC) and nucleus accumbens shell (NAcSh; Narita et al., 2006, 2007; Vittoz and Berridge, 2006; Vittoz et al., 2008), key brain structures of the neurocircuitry of addiction. The administration of OrxA directly in the VTA increased the motivation to self-administration cocaine, reflected by an increase in cocaine self-administration in discrete trials and under PR schedules of reinforcement but not under an FR schedule of reinforcement (Espana et al., 2011) as previously reported following intracerebroventricular OrxA administration (Boutrel et al., 2005). Altogether, these data confirm that the Orx system may not influence cocaine self-administration when conditions to obtain cocaine require low effort. Orexin inputs to the VTA appear to play a pivotal role in controlling cocaine intake when conditions to obtain the drug require a high level of motivation. Additional studies confirmed the importance of Orx transmission in the VTA during cocaine self-administration. Intra-VTA administration of the OrxR1 antagonist SB334867 reduced cocaine self-administration (Muschamp et al., 2014). These findings were subsequently extended to the central nucleus of the amygdala (CeA), showing that intra-CeA SB338467 administration decreased cocaine self-administration in rats under extended access conditions (Schmeichel et al., 2017; Figure 1).

### Alcohol

Similar to preclinical research on cocaine, there is extensive interest in investigating the effects of OrxR1 antagonism on alcohol intake. SB334867 has been shown to reduce alcohol intake under conditions that are characterized by high impulsivity (i.e., binge-like consumption; Olney et al., 2015). SB334867 decreased alcohol consumption and preference exclusively in rats that had high preference or motivation to drink alcohol (Moorman and Aston-Jones, 2009; Lei et al., 2016a; Moorman et al., 2017) and in genetically alcohol-preferring rats (Lawrence et al., 2006; Jupp et al., 2011). Moreover, OrxR1 blockade selectively decreased the escalation of alcohol drinking in mice that were alcohol-dependent, without altering lower levels of alcohol intake in non-dependent mice (Lopez et al., 2016).

In contrast, the blockade of OrxR2 with TCSOX229 did not alter alcohol intake (Lei et al., 2016a). However, other studies showed that OrxR2 blockade with subcutaneous JNJ10397049 administration dose-dependently reduced alcohol self-administration in rats (Shoblock et al., 2011). Intraperitoneal administration of the OrxR2 antagonist LSN2424100 lowered breakpoints on a PR schedule and reduced alcohol consumption in alcohol-preferring rats (Anderson et al., 2014). Corroborating these data, intracerebroventricular TCSOX229 administration reduced alcohol self-administration (Brown et al., 2013), confirming that OrxR2 signaling mediates some aspects of alcohol drinking. Other evidence suggests that the involvement of OrxR1 and OrxR2 activity in alcohol intake may be attributable to a general effect that is not specific to alcohol consumption. In fact, it was shown that blockade of both OrxRs reduced water intake (Anderson et al., 2014). Nevertheless, these data indicate that Orx neuropeptides *via* OrxR1 and OrxR2 signaling regulate the motivation for alcohol, and targeting OrxRs might be one approach to prevent compulsive, highly motivated behaviors that are directed toward alcohol drinking that is characteristic of alcohol use disorder.

Studies of central effects of Orx transmission on alcohol drinking have identified the involvement of the NAcSh, medial PFC (mPFC), CeA, and VTA (Lei et al., 2016b,^2019^; Olney et al., 2017). OrxR1 antagonism in the NAcSh was particularly effective in reducing high levels of alcohol drinking in mice, with no significant effect in mice that exhibited only a moderate level of drinking (Lei et al., 2019, 2020; Kwok et al., 2021). The blockade of OrxR2 with TCSOX229 in the VTA and CeA did not alter alcohol drinking (Olney et al., 2017), but TCSOX229 administration in the nucleus accumbens core (NAcC) decreased alcohol intake (Brown et al., 2013; Figure 1). Barson et al. (2015) reported that OrxA and OrxB administration in the anterior PVT (aPVT) but not pPVT increased alcohol intake, an effect that was reversed by the Orx2R antagonist TCSOX229 (Barson et al., 2015, 2017), supporting the hypothesis that the aPVT plays a role in mediating alcohol drinking *via* OrxR2 signaling (Figure 1). Overall, alcohol consumption data implicate Orx neuropeptides in the pathological motivation for alcohol drinking and demonstrate that both OrxR1 and OrxR2 play an important role in controlling voluntary alcohol consumption.

### Opioids

Several studies have investigated the involvement of OrxR antagonists in animal models of opioid use disorder. In the last two decades, the abuse of prescription opioids (e.g., oxycodone, remifentanil, and fentanyl) has received increasing attention because of the increase in the misuse of these medications and increase in overdoses (Seth et al., 2018). One of the first studies that tested the effects of OrxR antagonists on opioid self-administration showed that systemic SB334867 administration reduced heroin intake under both FR and PR schedules of reinforcement (Smith and Aston-Jones, 2012). OrxR2 antagonism with NBI80713 was subsequently shown to decrease heroin self-administration in rats that were given extended access to heroin (Schmeichel et al., 2015). However, when the effects of a different OrxR2 antagonist, TCSOX229, on oxycodone self-administration were tested under extended access conditions, no effect was observed (Matzeu and Martin-Fardon, 2020c). Systemic SB334867 administration significantly reduced oxycodone self-administration. Consistent with the efficacy of SB334867 in reducing oxycodone intake, other studies showed that this compound reduced the consumption of two other prescription opioids, remifentanil (Porter-Stransky et al., 2017) and fentanyl (Mohammadkhani et al., 2019, 2020). Interestingly, the efficacy of SB334867 in reducing remifentanil self-administration was preserved when the compound was injected in the ventral pallidum (VP; Mohammadkhani et al., 2019, 2020; Figure 1). These findings add to the growing body of evidence that targeting the Orx system may be beneficial for the treatment of opioid use disorder.

### Nicotine

Only limited data are available on the involvement of Orx neuropeptides in nicotine addiction. For example, Hollander et al. (2008) showed that systemic SB334867 administration or a site-specific injection of SB334867 in the insular cortex reduced nicotine self-administration (Figure 1) and abolished the stimulatory effects of nicotine on brain reward systems, reflected by reversal of the nicotine-induced lowering of intracranial self-stimulation thresholds. The blockade of OrxR1 appears to effectively reduce nicotine intake. Uslaner et al. (2014) reported that OrxR2 blockade had no effect on nicotine self-administration. Moreover, somatic signs of nicotine withdrawal were attenuated by the OrxR1 antagonist SB334867 but not by the OrxR2 antagonist TCSOX229 in mice and were also attenuated in *Orx* knockout mice (Plaza-Zabala et al., 2012). Additionally, local SB334867 infusion in the paraventricular nucleus of the hypothalamus decreased the expression of nicotine withdrawal (Plaza-Zabala et al., 2012). Overall, these data demonstrate that OrxR1 signaling plays a role in the reinforcing effects of nicotine and expression of nicotine withdrawal.

## Role of Orexin Neuropeptides in Drug Seeking

### Cocaine

For nearly two decades, our group and others have contributed significantly to characterizing the role of Orx neuropeptides in cocaine seeking. Intracerebroventricular OrxA administration led to the dose-dependent reinstatement of cocaine seeking without altering cocaine intake in rats, an effect that depended on corticotropin-releasing factor, suggesting that Orx and stress systems may closely interact to regulate cocaine-seeking behavior (Boutrel et al., 2005). Intracerebroventricular OrxA administration elevated intracranial self-stimulation thresholds, suggesting that OrxA negatively regulates the activity of brain reward circuitry. Furthermore, the selective OrxR1 receptor antagonist SB334867 blocked the stress-induced reinstatement of extinguished cocaine-seeking behavior (Boutrel et al., 2005; Zhou et al., 2012), confirming a role for Orx neuropeptides in driving drug seeking through the activation of stress pathways in the brain. When injected systemically, SB334867 but not the OrxR2 antagonist 4PT also reversed conditioned reinstatement that was induced by cocaine-related stimuli (Smith et al., 2009; Zhou et al., 2012; Martin-Fardon and Weiss, 2014a) but not reinstatement that was triggered by a priming injection of cocaine (Zhou et al., 2012). Another study found that pretreatment with SB334867 significantly attenuated cocaine-seeking behavior that was elicited by a drug-associated context following either extinction or abstinence (Smith et al., 2010), further demonstrating that OrxR1 signaling is critical for conditioned reinstatement. Altogether, these findings suggest that OrxR1 is necessary for cocaine seeking that is elicited by previously drug-paired cues/contexts and stress.

Studies have investigated discrete brain regions that are critically important for cocaine-seeking behavior. The PVT, especially the pPVT, was identified as one such key brain structure (Figure 1). A recent electrophysiological study that used a brain slice preparation for cellular recordings found that the superfusion of OrxA onto pPVT neurons increased the frequency of spontaneous excitatory postsynaptic currents (sEPSCs) but had no effect on miniature EPSCs (mEPSCs), suggesting a network-driven effect of OrxA. The amplitudes of s/mEPSCs were unaffected by OrxA, indicating a presynaptic action on glutamate release (Matzeu et al., 2018). Thus, the effect of OrxA in the pPVT appeared to be presynaptic and solely target glutamate release, with no involvement of postsynaptic efficacy. Microinjections of OrxA in the pPVT reinstated extinguished cocaine-seeking behavior, and this reinstatement was abolished by a concomitant injection of the OrxR2 antagonist TCSOX229 (Matzeu et al., 2016; Figure 1). Interestingly, this priming effect of OrxA in inducing cocaine-seeking behavior was not long lasting (Matzeu and Martin-Fardon, 2021), implying that the HYP (Orx)↔pPVT circuit undergoes neuroadaptive changes during abstinence, reflected by alterations of the efficacy of OrxA in inducing reinstatement when injected in the pPVT. In addition to the PVT, other brain regions that receive Orx inputs, such as the VTA and CeA, have been investigated because of their known role in cocaine-seeking behavior. Similar to observations in the pPVT, local injections of OrxA in the VTA reinstated cocaine-seeking behavior (Wang et al., 2009). However, in contrast to the pPVT, this priming effect of OrxA was reversed by a selective OrxR1 antagonist but not by an OrxR2 antagonist (Wang et al., 2009). Supporting the importance of OrxR1 signaling in the VTA during cocaine-seeking behavior (Figure 1), another study showed that SB334867 administration in the VTA attenuated the conditioned reinstatement of cocaine-seeking behavior, an effect that was not observed when the same OrxR1 antagonist was injected in the PVT (James et al., 2011). Finally, intra-CeA SB334867 administration significantly reduced the stress-induced reinstatement of cocaine-seeking behavior (Schmeichel et al., 2017). Together with findings in the VTA and pPVT, these results suggest the brain region-dependent involvement of OrxR1 vs. OrxR2 in cocaine-seeking behavior (e.g., OrxR1 in the VTA and CeA and OrxR2 in the pPVT; Figure 1).

### Alcohol

Several studies have shown that the Orx system is strongly engaged during alcohol-seeking behavior (e.g., Millan et al., 2010). OrxR1 blockade reduced alcohol cue-induced reinstatement (Lawrence et al., 2006; Martin-Fardon and Weiss, 2014b; Moorman et al., 2017), supporting the hypothesis that Orx neuropeptide transmission at OrxR1 specifically regulates high levels of motivation for alcohol. SB334867 has also been reported to effectively reduce the stress-induced reinstatement of alcohol-seeking behavior, but this effect was not specific for alcohol and instead generalized to the stress-induced reinstatement of sucrose seeking (Richards et al., 2008).

Investigations of specific brain regions where Orx neuropeptides are implicated in the control of alcohol-seeking behavior revealed that the pPVT plays a pivotal role in the stress-induced reinstatement of alcohol-seeking behavior (Matzeu and Martin-Fardon, 2020a; Figure 1). An intra-pPVT injection of the dual OrxR antagonist TCS1102 prevented stress-induced reinstatement selectively in alcohol-dependent rats (Matzeu and Martin-Fardon, 2020a). Another study showed that an injection of an OrxR2 antagonist in the NAcC did not affect alcohol seeking that was triggered by cue presentation (Brown et al., 2013). Similar to cocaine, these findings suggest that the involvement of OrxR1 vs. OrxR2 in alcohol-seeking behavior is brain region-dependent.

### Opioids

The pharmacological manipulation of OrxR1 as a potential treatment to prevent opioid relapse has received much research attention. SB334867 attenuated the reinstatement of heroin-seeking behavior that was elicited by discrete cues but not reinstatement that was elicited by a heroin priming injection (Smith and Aston-Jones, 2012). Several studies corroborated the efficacy of SB334867 in preventing prescription opioid seeking. For example, a recent study from our group found that systemic administration of the OrxR1 antagonist SB334867 but not OrxR2 TCSOX229 prevented the conditioned reinstatement of oxycodone-seeking behavior (Matzeu and Martin-Fardon, 2020c). Consistent with these findings, other studies of different opioids showed that OrxR1 blockade reduced the cue-induced reinstatement of remifentanil seeking (Porter-Stransky et al., 2017) and fentanyl seeking (Fragale et al., 2019). Studies that investigated the neurobiological basis of remifentanil seeking found that the VP is a key brain region where Orx neuropeptides that act through OrxR1 (Figure 1) can influence the cue-induced reinstatement of remifentanil seeking (Mohammadkhani et al., 2019, 2020).

### Nicotine

Limited and somehow controversial data are available on the effects of OrxR antagonists on nicotine-seeking behavior. Plaza-Zabala et al. (2013) showed that pretreatment with the OrxR1 antagonist SB334867 but not OrxR2 antagonist TCSOX229 decreased the cue-induced reinstatement of nicotine seeking. In contrast, Uslaner et al. (2014) showed that OrxR2 blockade significantly prevented nicotine seeking that was triggered by environmental cues. Specifically, the selective OrxR2 antagonist 2-SORA 18 reduced the cue-induced reinstatement of nicotine-seeking behavior but not drug-induced reinstatement (Uslaner et al., 2014). Although in apparent disagreement, these two studies demonstrate the importance of OrxR signaling for nicotine relapse.

## Restoring Normal Circadian Activity With Orexin Receptor Antagonists in Individuals With Substance Use Disorder

An estimated 5% of the world’s population uses drugs regularly, and nearly 0.6% suffer from a substance use disorder as defined by the *Diagnostic and Statistical Manual of Mental Disorders*, 5th edition (Merz, 2017). Drug abuse elicits detrimental consequences on a person’s overall well-being at the psychological, emotional, and social levels (Degenhardt and Hall, 2012). Sleep disturbances, including alterations of sleep architecture and the development of insomnia, are extremely common among people who suffer from substance use disorder (Wang and Teichtahl, 2007; Koob and Colrain, 2020; Brooks et al., 2021; Frers et al., 2021; Miller et al., 2021). Sleep disturbances during withdrawal and the early treatment of substance use disorder are a major problem across all classes of drugs of abuse and have been related to a failure of completing substance use disorder treatment (Wilkerson et al., 2021). The high incidence of insomnia symptoms and poor sleep quality, together with a lack of treatment completion, demonstrate that severe sleep disturbances are a major risk factor for relapse (Brower and Perron, 2010; Wilkerson et al., 2019).

Substance use disorder can also compromise a drug user’s nutrition and deleteriously affect dietary habits (Nabipour et al., 2014; Rush et al., 2016; Cusack et al., 2021; Mahboub et al., 2021). Individuals with drug addiction generally exhibit “unhealthy” lifestyles that consequently affect food intake, eventually leading to malnutrition or undernutrition (Wandler, 2003). Such individuals usually have irregular eating patterns, with unbalanced nutrient intake (Mahboub et al., 2021). During detoxification, they also have poor dietary habits, with low food consumption that is often caused by negative symptoms of abstinence from the drug (Mahboub et al., 2021). Notably, the frequency and duration of drug use can differentially influence the nutritional status of drug users (Santolaria-Fernandez et al., 1995), but further discussions of this aspect of drug use are beyond the scope of the present review.

Eating and the sleep/wake cycle are closely related to each another. Preclinical studies have shown that during food deprivation, animals exhibit shorter episodes of sleep, the frequency of which decreases as the length of deprivation increases (Borbely, 1977; Dewasmes et al., 1989), concomitant with an increase in locomotor activity (Moskowitz, 1959; Mabry and Campbell, 1975; Jones et al., 1990) that may be attributable to an increase in feeding behavior. Considering the crucial role of the Orx system in regulating feeding and sleeping, unsurprising is that dysfunction of the Orx system is related to disturbances in food intake and sleep. For example, individuals with narcolepsy who have low levels of Orx neuropeptides exhibit a concomitant increase in palatable food intake (van Holst et al., 2016), with a high incidence of binge eating (Dimitrova et al., 2011), suggesting that Orx peptides play a more intricate role than simply being “feeding peptides.”

To date, the literature indicates a role for Orx neuropeptides in mediating feeding behavior and sleep and regulating the motivation for drugs of abuse. As proposed for binge eating disorders (Mehr et al., 2021), we identify the Orx system as a potential neurobiological link between drugs of abuse and co-occurring eating and sleep dysregulations. We propose that pharmacotherapies that normalize Orx signaling, such as with OrxR antagonists, may effectively decrease the motivation for drugs of abuse and ameliorate sleep disturbances and unbalanced nutritional states that afflict individuals with substance use disorder. Overall, the preclinical studies discussed above demonstrate that OrxR1 may be a viable treatment target to selectively reduce craving and the motivation for drug intake. One common limitation of these studies, however, is that the efficacy of OrxR2 antagonism has not always been tested. Indeed, studies indicate that some aspects of addiction-related behaviors are also linked to OrxR2 signaling (e.g., Schmeichel et al., 2015; Matzeu et al., 2016), suggesting that OrxR2 might also play an important role in substance use disorder. Based on the assumption that both OrxR1 and OrxR2 play a role in drug addiction, the use of compounds that target both receptors might be beneficial for the treatment of substance use disorder.

The dual orexin receptor antagonist suvorexant (Belsomra™) is currently available for the clinical treatment of insomnia in the United States, Canada, and Japan. It is also gaining interest as a potential pharmacological treatment for addiction (Campbell et al., 2020a; James et al., 2020). The comorbidity of substance use disorder, sleep disruption, and malnutrition has been widely documented, and these conditions have been characterized by an imbalance in the Orx system. Thus, the use of suvorexant may be promising for restoring “normal” balance in the Orx system, mitigate relapse vulnerability, and reestablish “normal” physiological functions, including feeding and sleep. A clinical study of non-treatment-seeking subjects with cocaine use disorder who were treated with suvorexant reported promising results. Suvorexant improved sleep, reduced the response to acute stress, and reduced cocaine craving (Suchting et al., 2020). Another ongoing clinical study (Campbell et al., 2020b) is currently examining the efficacy of suvorexant for the treatment of comorbid alcohol use disorder and insomnia. These studies represent an important step toward the potential use of suvorexant for substance use disorder treatment and will provide valuable data to direct future clinical trials with other substances of abuse.

As mentioned above, drug addiction is often associated with dysregulations of eating, most commonly undernutrition. One concern could be that the use of OrxR antagonists that block Orx signaling might further suppress food intake, thus worsening undernutrition. Several studies have shown that OrxR1 blockade significantly decreased food intake and motivation toward food (e.g., Piccoli et al., 2012; Wiskerke et al., 2020; Freeman et al., 2021). However, other studies from our group showed that suppressing OrxR signaling had a minimal effect on food-directed behavior compared with drug-directed behavior (Martin-Fardon and Weiss, 2014a,b; Martin-Fardon et al., 2018; Schmeichel et al., 2018), suggesting that the use of an OrxR antagonist could have a minimal impact on food-directed behavior and be selective for the treatment of drug-directed behavior. Preclinical studies that examine the impact of OrxR antagonists on food-directed behavior in drug-dependent animals are lacking. Thus, the effect of manipulating the Orx system on an already compromised nutritional state in dependent subjects remains to be addressed.

## Conclusion and Future Directions

Orexin receptor antagonists are promising anti-addiction medications. Based on the considerable amount of data that have been generated by preclinical and clinical studies of the role of Orx neuropeptides in drug addiction, the National Institute on Drug Abuse, Division of Therapeutics and Medical Consequences, has recognized OrxR antagonists among medication development priorities for the treatment for addiction (Rasmussen et al., 2019). Although the data are very promising, several questions remain unanswered. For example, parallel findings on blood Orx levels in humans with substance use disorder (e.g., alcohol, Δ^9^-tetrahydrocannabinol, and nicotine) and preclinical studies of Orx in the brain remain counterintuitive. If the majority of preclinical studies show that OrxR antagonists that decrease Orx transmission can attenuate drug intake and drug-seeking behavior, human data show that stronger withdrawal symptoms are associated with lower blood levels of Orx, which are opposite to expectations (i.e., higher Orx levels rather than lower Orx levels). This suggests that brain and blood Orx levels might not follow the same dynamics. This issue remains unresolved and needs further investigation. Another issue is which OrxR antagonists (e.g., specific OrxR1 or OrxR2 antagonists or dual orexin receptor antagonists, such as suvorexant) are more effective as potential treatments for drug addiction. Additional unresolved issues are whether these compounds are effective across different classes of drugs and whether they are better suited in specific stages of the addiction cycle (Koob and Volkow, 2010; Koob, 2021). Knowing that the Orx system shows sexual dimorphism (Taheri et al., 1999; Johren et al., 2001), also unknown is whether the effects of these OrxR antagonists could be different depending on sex. Furthermore, studies need to characterize the bioavailability of these compounds, their toxicity, and the safety and efficacy of long-term treatment when used to treat comorbid substance use disorder and abnormal physiological functions, such as sleeping and feeding. The majority of preclinical studies have investigated only the acute effects of OrxR antagonists. The safety and efficacy of long-term treatment remain an important issue that needs to be addressed in preclinical studies. Lastly, investigations need to be extended to polysubstance use.

## Author Contributions

AM and RM-F wrote and reviewed the article. Both authors approved the final version.

## Conflict of Interest

The authors declare that the research was conducted in the absence of any commercial or financial relationships that could be construed as a potential conflict of interest.

## Publisher’s Note

All claims expressed in this article are solely those of the authors and do not necessarily represent those of their affiliated organizations, or those of the publisher, the editors and the reviewers. Any product that may be evaluated in this article, or claim that may be made by its manufacturer, is not guaranteed or endorsed by the publisher.
